# 4-[5-(4-Formyl­phen­oxy)pent­oxy]benzaldehyde

**DOI:** 10.1107/S1600536812034241

**Published:** 2012-08-08

**Authors:** Tomislav Balić, Berislav Marković, Ivana Balić

**Affiliations:** a Department of Chemistry, J. J. Strossmayer University, Osijek, Franje Kuhača 20, HR-31000 Osijek, Croatia

## Abstract

In the title compound, C_20_H_19_O_4_, the benzene rings, linked *via* five methyl­ene C atoms, form a dihedral angle of 77.28 (6)°. In the crystal, mol­ecules are linked *via* pairs of weak C—H⋯O inter­actions [graph set *R*_2_^2^(6)] into dimers that are further connected by additional weak C—H⋯O interactions [graph sets *R*_2_^2^(14), *R*_2_^2^(26) and *R*_2_^2^(6)].

## Related literature

For related structures and the synthesis of similar compounds, see: Ali *et al.* (2010 ▶); Dehno Khalaji *et al.* (2011 ▶); Han & Zhen (2005 ▶); Narasimha Moorthy *et al.* (2005 ▶). For the synthesis of Schiff bases and Schiff base complexes, see: Ma & Cao (2011 ▶); Ilhan *et al.* (2007 ▶); Keypour *et al.* (2008 ▶). For graph-set analysis, see: Bernstein *et al.* (1995 ▶).
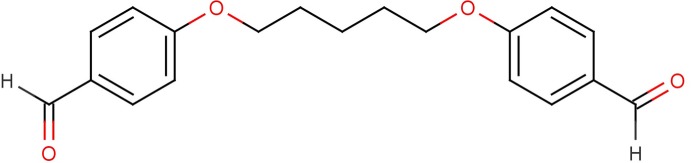


## Experimental

### 

#### Crystal data

C_19_H_20_O_4_
*M* *_r_* = 312.35
Monoclinic, 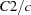

*a* = 22.3018 (8) Å
*b* = 4.6829 (16) Å
*c* = 31.6082 (12) Å
β = 103.752 (4)°
*V* = 3206.5 (11) Å^3^
*Z* = 8
Mo *K*α radiation
μ = 0.09 mm^−1^
*T* = 190 K
0.51 × 0.37 × 0.18 mm

#### Data collection

Oxford Diffraction Xcalibur Sapphire3 diffractometer
Absorption correction: multi-scan (*CrysAlis PRO*; Oxford Diffraction, 2009 ▶) *T*_min_ = 0.975, *T*_max_ = 1.000
9589 measured reflections
3136 independent reflections
2560 reflections with *I* > 2σ(*I*)
*R*_int_ = 0.020

#### Refinement

*R*[*F* ^2^ > 2σ(*F* ^2^)] = 0.039
*wR*(*F* ^2^) = 0.098
*S* = 1.06
3136 reflections
208 parameters
H-atom parameters constrained
Δρ_max_ = 0.19 e Å^−3^
Δρ_min_ = −0.18 e Å^−3^

### 

Data collection: *CrysAlis PRO* (Oxford Diffraction, 2009 ▶); cell refinement: *CrysAlis PRO*; data reduction: *CrysAlis PRO*; program(s) used to solve structure: *SIR2004* (Burla *et al.*, 2005 ▶); program(s) used to refine structure: *SHELXL97* (Sheldrick, 2008 ▶); molecular graphics: *ORTEP-3* (Farrugia, 1997 ▶); software used to prepare material for publication: *WinGX* (Farrugia, 1999 ▶), *PARST97* (Nardelli, 1995 ▶) and *Mercury* (Macrae *et al.*, 2006 ▶).

## Supplementary Material

Crystal structure: contains datablock(s) I, global. DOI: 10.1107/S1600536812034241/nc2286sup1.cif

Structure factors: contains datablock(s) I. DOI: 10.1107/S1600536812034241/nc2286Isup2.hkl

Supplementary material file. DOI: 10.1107/S1600536812034241/nc2286Isup3.cml

Additional supplementary materials:  crystallographic information; 3D view; checkCIF report

## Figures and Tables

**Table 1:** Hydrogen-bond geometry (Å, °)

*D*—H⋯*A*	*D*—H	H⋯*A*	*D*⋯*A*	*D*—H⋯*A*
C1—H1⋯O4^i^	0.95	2.53	3.3401 (18)	144
C8—H8*B*⋯O4^ii^	0.99	2.58	3.4815 (18)	152
C6—H6⋯O2^iii^	0.95	2.53	3.4487 (16)	163
C12—H12*B*⋯O1^iv^	0.99	2.50	3.4360 (18)	157
C14—H14⋯O3^v^	0.95	2.63	3.4977 (15)	151
C19—H19⋯O1^vi^	0.95	2.63	3.3698 (19)	135

Symmetry codes: (i) 
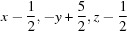
; (ii) 
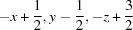
; (iii) 
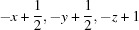
; (iv) 
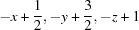
; (v) 
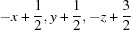
; (vi) 
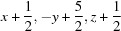
.

**Fig. 1.**
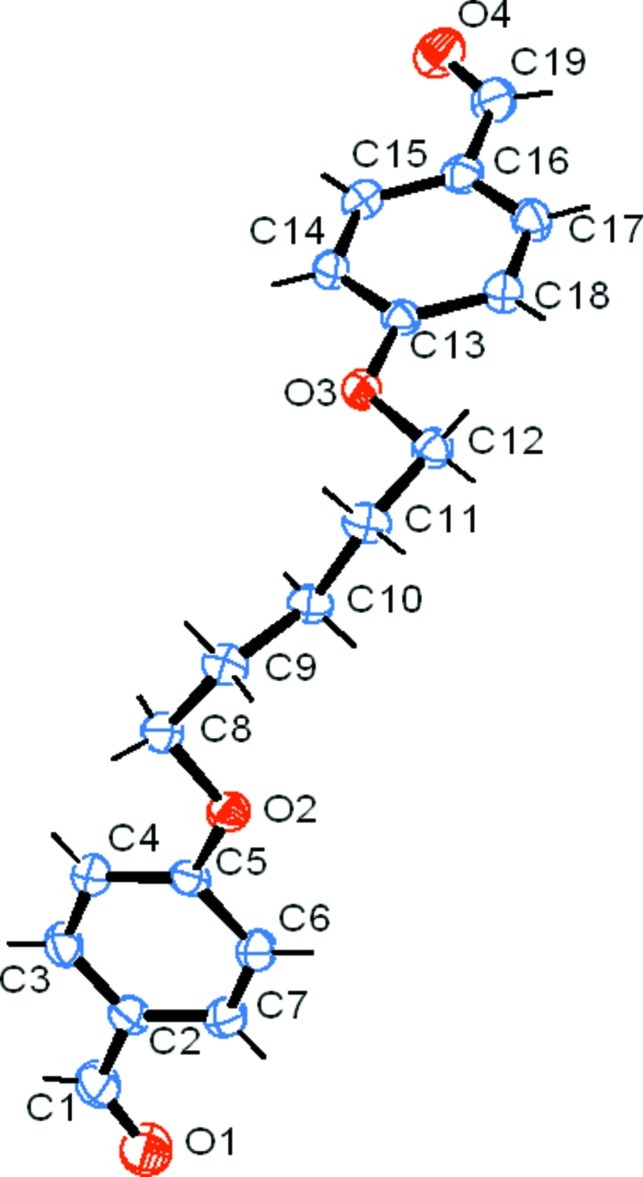
The molecular structure of the title compound with the atom numbering scheme. Displacement ellipsoids are drawn at the 50% probability level.

**Fig. 2.**
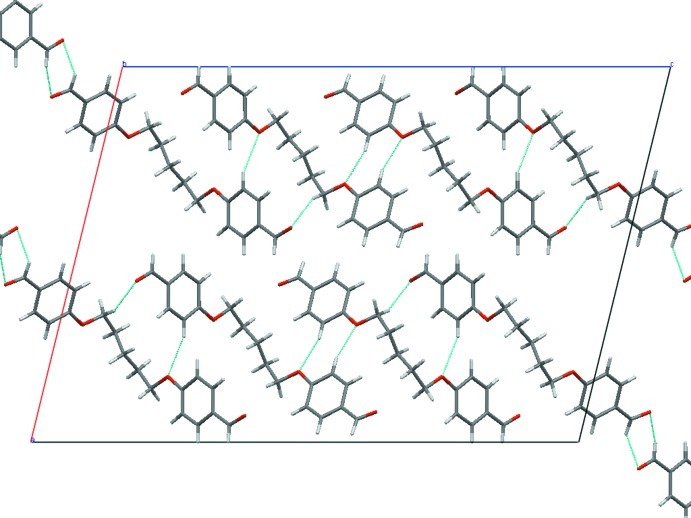
Crystal packing of title compound viewed down the b axis with dashed lines representing weak C—H···O intermolecular interactions.

## supplementary crystallographic information

### Comment

The aldehydes represent important class of organic compounds that are used for the condensation reaction with amines to form Schiff bases. Therefore, we have decided to explore the capability of novel dialdehydes, namely 4-[5-(4-formylphenoxy)pentoxy]benzaldehyde, as starting material for the synthesis of some novel Schiff bases. Dialdehydes have recently been investigated as valuble precursors for condensation reactions with amines (Ilhan *et al.* 2007; Ma & Cao 2011; Dehno Khalaji *et al.* 2011). Such condensation reactions can lead to the formation of macrocyclic ligands or complex compounds, by methods of template synthesis (Ilhan *et al.* 2007; Keypour *et al.* 2008; Ma & Cao 2011).

In the title molecule two formylphenoxy groups are linked by five methylene C atoms and the dihedral angle between benzen ring is 77.28° (Figure 1.). In the crystal, the molecules are linked into dimers *via* weak C—H···O hydrogen bonding into a staircase-like motif (Figure 2.). A similar motif in dialdehydes was observed by Narasimha Moorthy *et al.* (2005). Additional stabilization of the crystal structure is accomplished by a number of weak C— H···O hydrogen bonding interactions [graph set: *R*_2_^2^(14), *R*_2_^2^(26), *R*_2_^2^(6)] (Bernstein *et al.* 1995). O1 and O4 are involved in the formation of two different motifs: dimer formation [graph set *R*_2_^2^(6)] and ring formation [graph set *R*_2_^2^(26)]; specifically: O1···(C12— H12B, C19— H19) and O4···(C8— H8B, C1— H1) and thus making them bifurcated (Table 1).

### Experimental

*p-*hydroxybenzaldehyde (50 mmol) and K_2_CO_3_ (50 mmol) were mixed in 50 ml DMF and the mixture was brought to brisk reflux. 25 mmol of pentane-1,5-dibrom dissolved in 10 ml of DMF were added and the reaction mixture was refluxed for 4 h and stired at room temperature for additional 2 h. After the reaction was completed, 300 ml of demineralized water were added and the resulting percipitate was filtered and washed with plenty water. Single crystals suitable for X-ray diffraction were grown *via* liquid diffusion of water into 1,4-dioxane solution of title compound.

### Refinement

All H atoms were positioned geometrically and refined using a riding model with C—H = 0.93 - 0.97 Å and with *U*_iso_(H) = 1.2 times *U*_eq_(C).

### Crystal data

**Table:**

C_19_H_20_O_4_	*F*(000) = 1328
*M**_r_* = 312.35	*D*_x_ = 1.294 Mg m^−^^3^
Monoclinic, *C*2/*c*	Mo *K*α radiation, λ = 0.71073 Å
Hall symbol: -C 2yc	Cell parameters from 4466 reflections
*a* = 22.3018 (8) Å	θ = 4.3–28.4°
*b* = 4.6829 (16) Å	µ = 0.09 mm^−^^1^
*c* = 31.6082 (12) Å	*T* = 190 K
β = 103.752 (4)°	Block, colourless
*V* = 3206.5 (11) Å^3^	0.51 × 0.37 × 0.18 mm
*Z* = 8	

### Data collection

**Table:**

Oxford Diffraction Xcalibur Sapphire3 diffractometer	3136 independent reflections
Radiation source: Enhance (Mo) X-ray Source	2560 reflections with *I* > 2σ(*I*)
Graphite monochromator	*R*_int_ = 0.020
ω scans	θ_max_ = 26.0°, θ_min_ = 4.3°
Absorption correction: multi-scan (*CrysAlis PRO*; Oxford Diffraction, 2009)	*h* = −24→27
*T*_min_ = 0.975, *T*_max_ = 1.000	*k* = −5→5
9589 measured reflections	*l* = −38→37

### Refinement

**Table:**

Refinement on *F*^2^	Primary atom site location: structure-invariant direct methods
Least-squares matrix: full	Secondary atom site location: difference Fourier map
*R*[*F*^2^ > 2σ(*F*^2^)] = 0.039	Hydrogen site location: inferred from neighbouring sites
*wR*(*F*^2^) = 0.098	H-atom parameters constrained
*S* = 1.06	*w* = 1/[σ^2^(*F*_o_^2^) + (0.0389*P*)^2^ + 1.7845*P*] where *P* = (*F*_o_^2^ + 2*F*_c_^2^)/3
3136 reflections	(Δ/σ)_max_ = 0.001
208 parameters	Δρ_max_ = 0.19 e Å^−^^3^
0 restraints	Δρ_min_ = −0.18 e Å^−^^3^

### Special details

**Table:**

Geometry. All e.s.d.'s (except the e.s.d. in the dihedral angle between two l.s. planes) are estimated using the full covariance matrix. The cell e.s.d.'s are taken into account individually in the estimation of e.s.d.'s in distances, angles and torsion angles; correlations between e.s.d.'s in cell parameters are only used when they are defined by crystal symmetry. An approximate (isotropic) treatment of cell e.s.d.'s is used for estimating e.s.d.'s involving l.s. planes.
Refinement. Refinement of *F*^2^ against ALL reflections. The weighted *R*-factor *wR* and goodness of fit *S* are based on *F*^2^, conventional *R*-factors *R* are based on *F*, with *F* set to zero for negative *F*^2^. The threshold expression of *F*^2^ > σ(*F*^2^) is used only for calculating *R*-factors(gt) *etc*. and is not relevant to the choice of reflections for refinement. *R*-factors based on *F*^2^ are statistically about twice as large as those based on *F*, and *R*- factors based on ALL data will be even larger.

### Fractional atomic coordinates and isotropic or equivalent isotropic displacement parameters (Å^2^)

**Table:**

	*x*	*y*	*z*	*U*_iso_*/*U*_eq_	
O1	0.08040 (5)	1.1018 (3)	0.38389 (4)	0.0547 (3)	
O2	0.18750 (4)	0.2636 (2)	0.54219 (3)	0.0319 (2)	
O3	0.32553 (4)	0.2744 (2)	0.72116 (3)	0.0285 (2)	
O4	0.42945 (5)	1.1213 (2)	0.87886 (3)	0.0477 (3)	
C1	0.06143 (7)	1.0219 (3)	0.41477 (5)	0.0405 (4)	
H1	0.0226	1.0931	0.4173	0.049*	
C2	0.09356 (6)	0.8247 (3)	0.44835 (4)	0.0304 (3)	
C3	0.06566 (6)	0.7310 (3)	0.48055 (5)	0.0338 (3)	
H3	0.0254	0.7972	0.4806	0.041*	
C4	0.09498 (6)	0.5429 (3)	0.51280 (4)	0.0312 (3)	
H4	0.0752	0.4815	0.5347	0.037*	
C5	0.15385 (6)	0.4457 (3)	0.51250 (4)	0.0268 (3)	
C6	0.18249 (6)	0.5389 (3)	0.48024 (4)	0.0330 (3)	
H6	0.2227	0.4723	0.4800	0.040*	
C7	0.15283 (6)	0.7264 (3)	0.44882 (4)	0.0341 (3)	
H7	0.1728	0.7901	0.4272	0.041*	
C8	0.16073 (6)	0.1576 (3)	0.57633 (4)	0.0299 (3)	
H8A	0.1226	0.0486	0.5637	0.036*	
H8B	0.1500	0.3185	0.5934	0.036*	
C9	0.20774 (6)	−0.0332 (3)	0.60517 (4)	0.0298 (3)	
H9A	0.2207	−0.1820	0.5869	0.036*	
H9B	0.1880	−0.1306	0.6262	0.036*	
C10	0.26483 (6)	0.1251 (3)	0.63036 (4)	0.0283 (3)	
H10A	0.2517	0.2899	0.6456	0.034*	
H10B	0.2878	0.2004	0.6095	0.034*	
C11	0.30789 (6)	−0.0631 (3)	0.66370 (4)	0.0301 (3)	
H11A	0.2830	−0.1720	0.6803	0.036*	
H11B	0.3282	−0.2025	0.6481	0.036*	
C12	0.35692 (6)	0.1019 (3)	0.69529 (4)	0.0285 (3)	
H12A	0.3861	−0.0305	0.7141	0.034*	
H12B	0.3803	0.2246	0.6794	0.034*	
C13	0.35974 (6)	0.4398 (3)	0.75307 (4)	0.0247 (3)	
C14	0.32629 (6)	0.6109 (3)	0.77544 (4)	0.0280 (3)	
H14	0.2825	0.6047	0.7680	0.034*	
C15	0.35664 (6)	0.7880 (3)	0.80814 (4)	0.0300 (3)	
H15	0.3336	0.9049	0.8231	0.036*	
C16	0.42127 (6)	0.7984 (3)	0.81970 (4)	0.0301 (3)	
C17	0.45393 (6)	0.6280 (3)	0.79728 (4)	0.0320 (3)	
H17	0.4978	0.6341	0.8048	0.038*	
C18	0.42406 (6)	0.4486 (3)	0.76408 (4)	0.0296 (3)	
H18	0.4471	0.3330	0.7490	0.036*	
C19	0.45393 (7)	0.9823 (3)	0.85514 (5)	0.0378 (4)	
H19	0.4976	0.9940	0.8600	0.045*	

### Atomic displacement parameters (Å^2^)

**Table:**

	*U*^11^	*U*^22^	*U*^33^	*U*^12^	*U*^13^	*U*^23^
O1	0.0500 (7)	0.0681 (8)	0.0458 (7)	0.0129 (6)	0.0108 (5)	0.0243 (6)
O2	0.0310 (5)	0.0398 (6)	0.0251 (5)	0.0092 (4)	0.0069 (4)	0.0049 (4)
O3	0.0253 (5)	0.0331 (5)	0.0267 (5)	−0.0014 (4)	0.0056 (4)	−0.0033 (4)
O4	0.0512 (6)	0.0523 (7)	0.0426 (6)	−0.0169 (6)	0.0171 (5)	−0.0155 (5)
C1	0.0343 (8)	0.0446 (9)	0.0405 (8)	0.0074 (7)	0.0045 (7)	0.0073 (7)
C2	0.0290 (7)	0.0312 (7)	0.0286 (7)	0.0024 (6)	0.0024 (5)	−0.0014 (6)
C3	0.0246 (7)	0.0385 (8)	0.0370 (8)	0.0056 (6)	0.0045 (6)	−0.0006 (6)
C4	0.0275 (7)	0.0363 (8)	0.0303 (7)	0.0016 (6)	0.0080 (6)	0.0005 (6)
C5	0.0279 (6)	0.0285 (7)	0.0219 (6)	0.0029 (6)	0.0016 (5)	−0.0030 (5)
C6	0.0284 (7)	0.0407 (8)	0.0305 (7)	0.0088 (6)	0.0080 (6)	0.0011 (6)
C7	0.0335 (7)	0.0411 (8)	0.0285 (7)	0.0047 (6)	0.0090 (6)	0.0030 (6)
C8	0.0301 (7)	0.0325 (7)	0.0270 (7)	−0.0021 (6)	0.0065 (6)	−0.0006 (6)
C9	0.0334 (7)	0.0266 (7)	0.0278 (7)	−0.0009 (6)	0.0042 (6)	0.0005 (5)
C10	0.0331 (7)	0.0256 (7)	0.0251 (7)	−0.0005 (6)	0.0048 (6)	0.0008 (5)
C11	0.0362 (7)	0.0268 (7)	0.0255 (7)	0.0010 (6)	0.0035 (6)	0.0004 (5)
C12	0.0294 (7)	0.0298 (7)	0.0267 (7)	0.0035 (6)	0.0074 (5)	0.0015 (6)
C13	0.0262 (6)	0.0257 (7)	0.0216 (6)	−0.0024 (5)	0.0043 (5)	0.0043 (5)
C14	0.0241 (6)	0.0325 (7)	0.0277 (7)	−0.0011 (6)	0.0065 (5)	0.0031 (6)
C15	0.0333 (7)	0.0303 (7)	0.0283 (7)	−0.0002 (6)	0.0113 (6)	0.0006 (6)
C16	0.0332 (7)	0.0299 (7)	0.0271 (7)	−0.0055 (6)	0.0069 (6)	0.0023 (6)
C17	0.0255 (7)	0.0356 (8)	0.0332 (7)	−0.0039 (6)	0.0038 (6)	0.0013 (6)
C18	0.0266 (6)	0.0319 (7)	0.0306 (7)	0.0003 (6)	0.0076 (6)	0.0007 (6)
C19	0.0370 (8)	0.0405 (8)	0.0360 (8)	−0.0114 (7)	0.0092 (7)	−0.0035 (7)

### Geometric parameters (Å, º)

**Table:**

O1—C1	1.2118 (18)	C9—H9A	0.9900
O2—C5	1.3552 (15)	C9—H9B	0.9900
O2—C8	1.4401 (15)	C10—C11	1.5261 (17)
O3—C13	1.3541 (15)	C10—H10A	0.9900
O3—C12	1.4436 (15)	C10—H10B	0.9900
O4—C19	1.2160 (18)	C11—C12	1.5069 (18)
C1—C2	1.4596 (19)	C11—H11A	0.9900
C1—H1	0.9500	C11—H11B	0.9900
C2—C3	1.3847 (19)	C12—H12A	0.9900
C2—C7	1.3963 (19)	C12—H12B	0.9900
C3—C4	1.3876 (19)	C13—C18	1.3938 (17)
C3—H3	0.9500	C13—C14	1.3962 (18)
C4—C5	1.3915 (18)	C14—C15	1.3717 (18)
C4—H4	0.9500	C14—H14	0.9500
C5—C6	1.3957 (19)	C15—C16	1.4009 (18)
C6—C7	1.3719 (19)	C15—H15	0.9500
C6—H6	0.9500	C16—C17	1.3838 (19)
C7—H7	0.9500	C16—C19	1.4626 (19)
C8—C9	1.5081 (18)	C17—C18	1.3850 (19)
C8—H8A	0.9900	C17—H17	0.9500
C8—H8B	0.9900	C18—H18	0.9500
C9—C10	1.5241 (18)	C19—H19	0.9500
			
C5—O2—C8	118.40 (10)	C11—C10—H10A	109.0
C13—O3—C12	118.60 (10)	C9—C10—H10B	109.0
O1—C1—C2	125.17 (14)	C11—C10—H10B	109.0
O1—C1—H1	117.4	H10A—C10—H10B	107.8
C2—C1—H1	117.4	C12—C11—C10	113.55 (11)
C3—C2—C7	118.51 (13)	C12—C11—H11A	108.9
C3—C2—C1	120.40 (12)	C10—C11—H11A	108.9
C7—C2—C1	121.08 (13)	C12—C11—H11B	108.9
C2—C3—C4	121.69 (12)	C10—C11—H11B	108.9
C2—C3—H3	119.2	H11A—C11—H11B	107.7
C4—C3—H3	119.2	O3—C12—C11	106.81 (10)
C3—C4—C5	118.91 (13)	O3—C12—H12A	110.4
C3—C4—H4	120.5	C11—C12—H12A	110.4
C5—C4—H4	120.5	O3—C12—H12B	110.4
O2—C5—C4	124.71 (12)	C11—C12—H12B	110.4
O2—C5—C6	115.36 (11)	H12A—C12—H12B	108.6
C4—C5—C6	119.92 (12)	O3—C13—C18	124.55 (12)
C7—C6—C5	120.23 (12)	O3—C13—C14	115.54 (11)
C7—C6—H6	119.9	C18—C13—C14	119.91 (12)
C5—C6—H6	119.9	C15—C14—C13	120.08 (12)
C6—C7—C2	120.73 (13)	C15—C14—H14	120.0
C6—C7—H7	119.6	C13—C14—H14	120.0
C2—C7—H7	119.6	C14—C15—C16	120.69 (13)
O2—C8—C9	107.74 (10)	C14—C15—H15	119.7
O2—C8—H8A	110.2	C16—C15—H15	119.7
C9—C8—H8A	110.2	C17—C16—C15	118.72 (12)
O2—C8—H8B	110.2	C17—C16—C19	120.32 (12)
C9—C8—H8B	110.2	C15—C16—C19	120.96 (13)
H8A—C8—H8B	108.5	C16—C17—C18	121.38 (12)
C8—C9—C10	113.70 (11)	C16—C17—H17	119.3
C8—C9—H9A	108.8	C18—C17—H17	119.3
C10—C9—H9A	108.8	C17—C18—C13	119.22 (12)
C8—C9—H9B	108.8	C17—C18—H18	120.4
C10—C9—H9B	108.8	C13—C18—H18	120.4
H9A—C9—H9B	107.7	O4—C19—C16	124.93 (14)
C9—C10—C11	112.96 (11)	O4—C19—H19	117.5
C9—C10—H10A	109.0	C16—C19—H19	117.5
			
O1—C1—C2—C3	−175.26 (16)	C9—C10—C11—C12	−167.57 (11)
O1—C1—C2—C7	4.8 (2)	C13—O3—C12—C11	178.44 (10)
C7—C2—C3—C4	−0.2 (2)	C10—C11—C12—O3	65.94 (14)
C1—C2—C3—C4	179.78 (13)	C12—O3—C13—C18	−2.50 (17)
C2—C3—C4—C5	−0.3 (2)	C12—O3—C13—C14	177.13 (11)
C8—O2—C5—C4	0.99 (19)	O3—C13—C14—C15	−179.49 (11)
C8—O2—C5—C6	−179.73 (11)	C18—C13—C14—C15	0.16 (19)
C3—C4—C5—O2	179.59 (12)	C13—C14—C15—C16	−0.43 (19)
C3—C4—C5—C6	0.3 (2)	C14—C15—C16—C17	0.5 (2)
O2—C5—C6—C7	−179.25 (12)	C14—C15—C16—C19	−178.70 (13)
C4—C5—C6—C7	0.1 (2)	C15—C16—C17—C18	−0.2 (2)
C5—C6—C7—C2	−0.6 (2)	C19—C16—C17—C18	178.94 (13)
C3—C2—C7—C6	0.7 (2)	C16—C17—C18—C13	0.0 (2)
C1—C2—C7—C6	−179.35 (14)	O3—C13—C18—C17	179.69 (12)
C5—O2—C8—C9	−178.73 (11)	C14—C13—C18—C17	0.07 (19)
O2—C8—C9—C10	66.89 (14)	C17—C16—C19—O4	−173.80 (14)
C8—C9—C10—C11	172.47 (11)	C15—C16—C19—O4	5.4 (2)

### Hydrogen-bond geometry (Å, º)

**Table:**

*D*—H···*A*	*D*—H	H···*A*	*D*···*A*	*D*—H···*A*
C1—H1···O4^i^	0.95	2.53	3.3401 (18)	144
C8—H8*B*···O4^ii^	0.99	2.58	3.4815 (18)	152
C6—H6···O2^iii^	0.95	2.53	3.4487 (16)	163
C12—H12*B*···O1^iv^	0.99	2.50	3.4360 (18)	157
C14—H14···O3^v^	0.95	2.63	3.4977 (15)	151
C19—H19···O1^vi^	0.95	2.63	3.3698 (19)	135

Symmetry codes: (i) *x*−1/2, −*y*+5/2, *z*−1/2; (ii) −*x*+1/2, *y*−1/2, −*z*+3/2; (iii) −*x*+1/2, −*y*+1/2, −*z*+1; (iv) −*x*+1/2, −*y*+3/2, −*z*+1; (v) −*x*+1/2, *y*+1/2, −*z*+3/2; (vi) *x*+1/2, −*y*+5/2, *z*+1/2.
